# Bis-Bibenzyls from the Liverwort *Pellia endiviifolia* and Their Biological Activity

**DOI:** 10.3390/plants10061063

**Published:** 2021-05-26

**Authors:** Ivana Ivković, Miroslav Novaković, Milan Veljić, Marija Mojsin, Milena Stevanović, Petar D. Marin, Danka Bukvički

**Affiliations:** 1 Institute of Botany and Botanical Garden “Jevremovac”, Faculty of Biology, University of Belgrade, 11000 Begrade, Serbia; ivkovicivana@yahoo.com (I.I.); veljicm@bio.bg.ac.rs (M.V.); pdmarin@bio.bg.ac.rs (P.D.M.); 2 Department of Chemistry, Institute of Chemistry, Technology and Metallurgy, University of Belgrade, 11000 Belgrade, Serbia; mironov76@yahoo.com; 3 Institute of Molecular Genetics and Genetic Engineering, University of Belgrade, 11042 Belgrade, Serbia; mojsin@imgge.bg.ac.rs (M.M.); milenastevanovic@imgge.bg.ac.rs (M.S.); 4 Faculty of Biology, University of Belgrade, 11000 Belgrade, Serbia; 5 Serbian Academy of Sciences and Arts, 11001 Belgrade, Serbia

**Keywords:** *Pellia endiviifolia*, perrottetin E derivatives, 1D and 2D NMR, human leukemia cell lines, human teratocarcinoma cell line, human glioblastoma cell line

## Abstract

Based on previous investigations where bis-bibenzyls isolated from liverworts showed various biological activities (cytotoxic, antimicrobial, and antiviral), we investigated their cytotoxic activity in several human cancer cell lines. From the methylene-chloride/methanol extract of the liverwort *Pellia endiviifolia*, three bis-bibenzyls of the perrottetin type were isolated, namely perrottetin E, 10′-hydroxyperrottetin E, and 10,10′-dihydroxyperrottetin E. The last two were found for the first time in this species. Their structures were resolved using 1D and 2D NMR, as well as by comparison with data in the literature. Cytotoxic activity of the isolated compounds was tested on three human leukemia cell lines, HL-60 (acute promyelocytic leukemia cells), U-937 (acute monocytic leukemia cells), and K-562 (human chronic myelogenous leukemia cells), as well as on human embryonal teratocarcinoma cell line (NT2/D1) and human glioblastoma cell lines A-172 and U-251, and compared to the previously isolated bis-bibenzyls (perrottetins) of similar structure. The isolated compounds exhibited modest activity against leukemia cells and significant activity against NT2/D1 and A-172. Overall, the most active cytotoxic compounds in this investigation were perrottetin E (1), isolated in this work from *Pellia endiviifolia*, and perrottetin F phenanthrene derivative (7), previously isolated from *Lunularia cruciata* and added for a comparison of their cytotoxic activity.

## 1. Introduction

Bryophytes—small, non-vascular plants—are usually divided into three separate divisions: Marchantiophyta (liverworts), Bryophyta (genuine mosses), and Anthocerotophyta (hornworts) [1]. Based on morphological characteristics, liverworts are classified into thalloid and leafy species. In comparison with the other two divisions, liverworts possess oil bodies in their cells that carry various secondary metabolites—mostly aromatic compounds, acetogenins, and terpenoids [2,3]. Different types of acyclic and cyclic bis-bibenzyls, along with their derivatives, are the most characteristic compounds isolated from the liverworts. Phenolic bis-bibenzyls, with four aromatic rings, derive from the bibenzyl lunularin or its precursor lunularic acid [4]. A large number of bryophytes is highly used as medicinal plants in Chinese and Indian traditional medicine to cure various skin diseases, such as wounds, bruises, and burns [5,6]. Secondary metabolites produced by liverworts show interesting biological activities, such as cytotoxic, antibacterial, antifungal, antiviral, and antioxidant [7,8]. They also possess muscle relaxing, tubulin inhibitory, nitric oxide production inhibitory, and calcium inhibitory activities [9]. *Pellia endiviifolia* (Dicks.) Dumort. (Pelliaceae) is a thalloid, dioicous liverwort that is broadly distributed in the Northern Hemisphere [10]. This hydrophilous species could be found by watercourses, on stones, in shaded ditches, and moist soil. Previous chemical analysis of *P. endiviifolia* has shown the presence of three bis-bibenzyl ethers: perrottetin E, perrottetin E-11′-methyl ether, and 14-hydroxyperrottetin E-11′-methyl ether [11]. Different types of sacculatane diterpenoids, such as sacculatal, isosacculatal, sacculatanolide, pellianolactol, etc., have also been isolated from *P. endiviifolia* [12]. The cytotoxic effect of liverworts’ secondary metabolites, e.g., sacculatal, is known from the literature [13,14]. In the previous investigation, bis-bibenzyl perrottetin E isolated from *Radula perrottetii* showed cytotoxic effect on human nasopharyngeal epidermoid carcinoma (KB) cells [15], as well as inhibition of thrombin activation [16]. In this paper, the isolation and the structure investigation of the chemical constituents originated from *Pellia endiviifolia* are presented. Cytotoxic activity of the isolated compounds was tested on three human leukemia, one human embryonal teratocarcinoma, and two human glioblastoma cell lines, and compared to previously isolated bis-bibenzyls of similar structure [17,18]. All these results show the important anticancer role of the perrottetin-like compounds on the tested cell lines.

## 2. Results

### Isolation of Bis-Bibenzyls and Cytotoxic Activity

**Figure 1:**
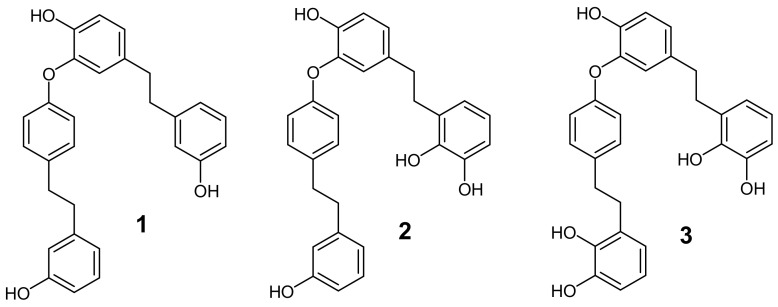
Perrottetin-type bis-bibenzyls isolated from *P. endiviifolia*.

From the *P. endiviifolia* CH_2_Cl_2_/MeOH (2:1) extract, three bis-bibenzyls of the perrottetin type were isolated (Figure 1). Perrottetin E (1) was already a known constituent from this liverwort (11), whereas 10′-hydroxyperrottetin E (2) and 10,10′-dihydroxyperrottetin E (3) were known compounds isolated from *Pellia epiphylla* [19] but in this work, they were isolated for the first time from *P. endiviifolia*.

**Figure 2:**
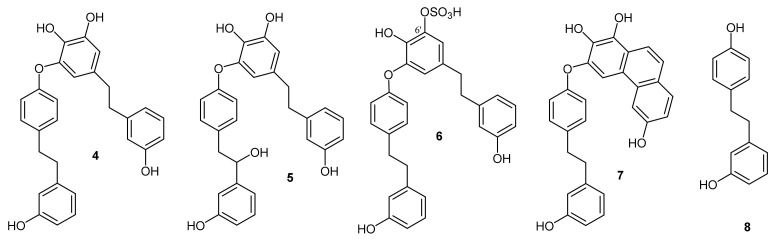
Perrottetin-type bis-bibenzyls added for comparative investigation of cytotoxic activity.

For the analysis of the biological activity, additional perrottetin-type compounds (4–7) and bibenzyl lunularin (8) isolated in previous works [17,19] were added to the isolated compounds 1, 2, and 3: perrottetin F (4), 8-hydroxyperrottetin F (5), perrottetin F-6′-sulphate (6), 2,3,7-trihydroxy-4-(3′-hydroxy-1-bibenzyloxy)-phenanthrene (7), and lunularin (8) (Figure 2).

**Figure 3:**
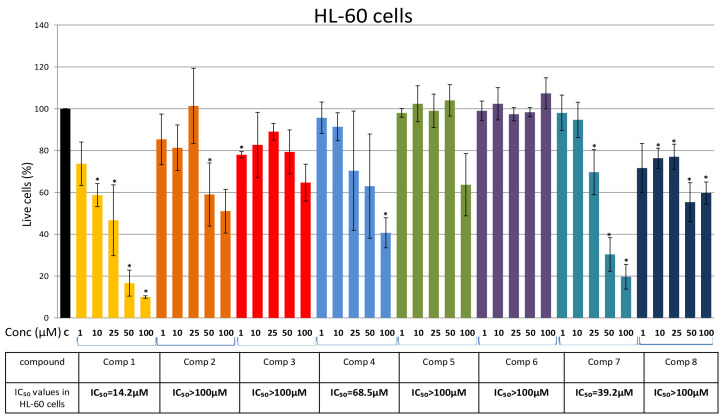
Effects of Compounds 1–8 on HL-60 cells. Cells were treated for 48 h with increasing concentrations (1 μM, 10 μM, 25 μM, 50 μM, and 100 μM) of the tested compounds and cell viability was assessed using an MTS assay and expressed as the percentage of absorbance relative to the vehicle control (HL-60 cells treated with DMSO (dimethyl sulfoxide)), which was set at 100%. Data are presented as the mean ± SEM of at least three independent experiments. An asterisk denotes a significant difference from the control (* *p* < 0.05). SEM—standard error of the mean.

**Figure 4:**
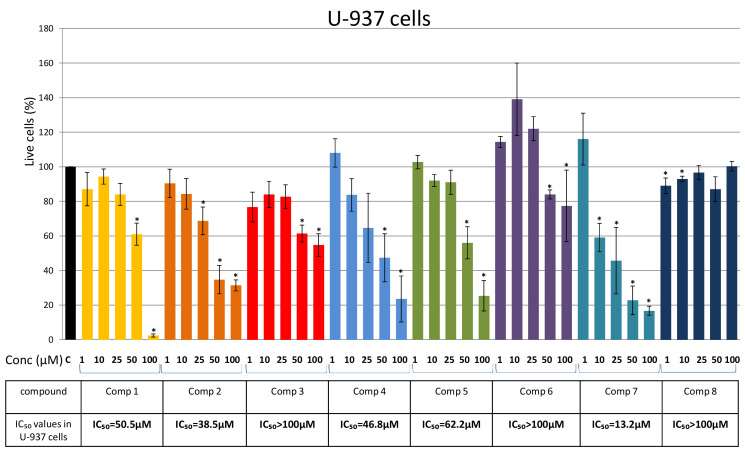
Effects of Compounds 1–8 on U-937 cells. Cells were treated for 48 h with increasing concentrations (1 μM, 10 μM, 25 μM, 50 μM, and 100 μM) of the tested compounds and cell viability was assessed using an MTS assay and expressed as the percentage of absorbance relative to the vehicle control (U-937 cells treated with DMSO), which was set at 100%. Data are presented as the mean ± SEM of at least three independent experiments. An asterisk denotes a significant difference from the control (* *p* < 0.05). SEM—standard error of the mean.

**Figure 5:**
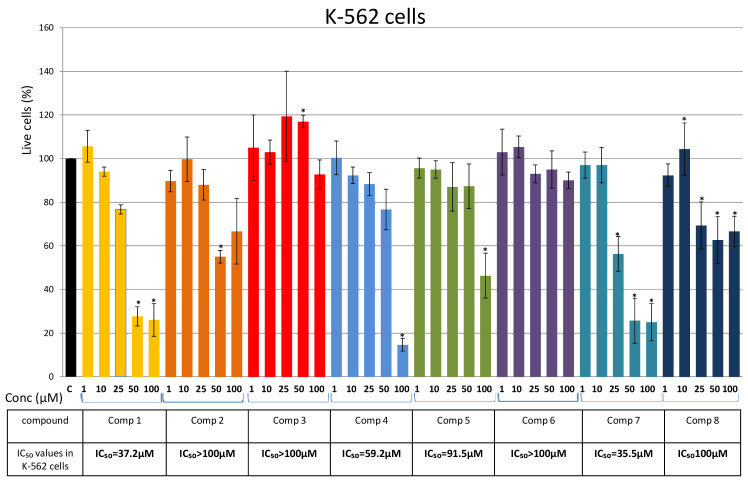
Effects of Compounds 1–8 on K-562 cells. Cells were treated for 48 h with increasing concentrations (1 μM, 10 μM, 25 μM, 50 μM, and 100 μM) of the tested compounds and cell viability was assessed using an MTS assay and expressed as the percentage of absorbance relative to the vehicle control (K-562 cells treated with DMSO), which was set at 100%. Data are presented as the mean ± SEM of at least three independent experiments. An asterisk denotes a significant difference from the control (* *p* < 0.05). SEM—standard error of the mean.

First, we analyzed the cytotoxic effects of Compounds 1–8 on acute (HL-60 and U-937) and chronic (K-562) myeloid leukemia cell lines. Cells were treated for 48 h with increasing concentrations of the selected compounds (1 μM, 10 μM, 25 μM, 50 μM, and 100 μM) and cell viability was evaluated using an MTS [3-(4,5-dimethylthiazol-2-yl)-5-(3-carboxymethoxyphenyl)-2-(4-sulfophenyl)-2H-tetrazolium)] assay (Figure 3, Figure 4 and Figure 5). As presented in Figure 3, with IC_50_ values of 14.2 μM and 39.2 μM, Compounds 1 and 7 had the highest cytotoxicity on HL-60 cells, respectively. Compound 4 showed a weak cytotoxic effect (IC_50_ = 68.5 μM), while the other tested compounds, including Compounds 2 and 3 for the first time isolated from *P. endiviifolia*, showed non-cytotoxicity toward HL-60 (IC_50_ values > 100 μM) (Figure 3). We observed a high standard error of the mean (SEM) for the cytotoxic activity of Compound 1 (at a concentration of 25 μM) and Compounds 2 and 4 (at concentrations 25 μM and 50 μM) (Figure 3). We speculate that HL-60 cellular heterogeneity [20] (reviewed in the use of the HL-60 cell line to measure the opsonic capacity of pneumococcal antibodies) contributes to the observed differences in the sensitivity to the tested compounds.

U-937 cells showed slightly higher sensitivity to the selected compounds (Figure 4). Compound 7 had the greatest effect on U-937 cells with an IC_50_ of 13.2 μM, whereas Compounds 4, 1, and 5 showed a moderate to weak effect, with IC_50_ values of 46.8 μM, 50.5 μM, and 62.2 μM, respectively (Figure 4). Regarding the compounds isolated for the first time from *P. endiviifolia*, Compound 2 exhibited strong cytotoxicity, with an IC_50_ of 38.5 μM, while Compound 3 showed no cytotoxic effect against U-937 cells (Figure 4).

Chronic myeloid leukemia K-562 cells’ responsiveness to the tested compounds were comparable with the results obtained in HL-60 and U937 (Figure 5). The calculated IC_50_ values for Compounds 7, 1, and 4 were 35.5 µM, 37.2 µM, and 59.2 µM, respectively (Figure 5). The percentage of cell viability after treatment with the other tested compounds, including Compounds 2 and 3 tested here for the first time, showed non-cytotoxicity (IC_50_ between 90 µM and 100 µM) (Figure 5).

The presented results show that Compounds 1–8 have similar and comparable effects on the viability of AML and CML cells. The highest cytotoxic effect on U-937 and K-562 was induced by Compound 7 (Figure 4 and Figure 5), while Compound 1 showed the highest cytotoxicity against HL-60 cells (Figure 3). Results obtained with the compounds isolated for the first time from *P. endiviifolia* showed strong cytotoxic activity of Compound 2 against U-937 cells.

**Figure 6:**
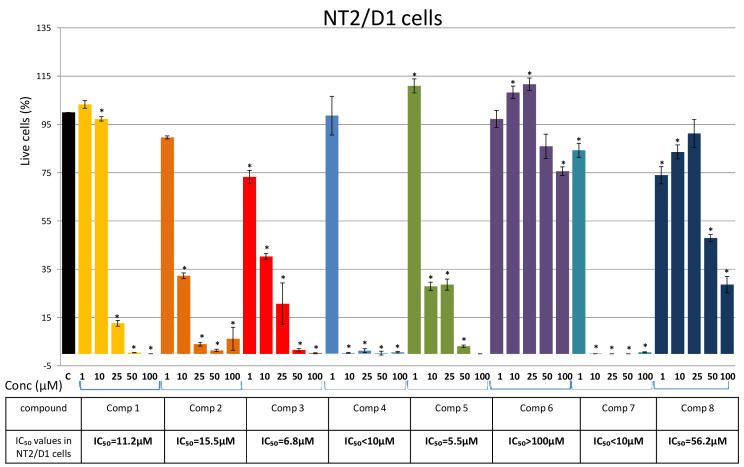
Effects of Compounds 1–8 on NT2/D1 cells. Cells were treated for 48 h with increasing concentrations (1 μM, 10 μM, 25 μM, 50 μM, and 100 μM) of the tested compounds and cell viability was assessed using an MTT assay and expressed as the percentage of absorbance relative to the vehicle control (NT2/D1 cells treated with DMSO), which was set at 100%. Data are presented as the mean ± SEM of at least three independent experiments. An asterisk denotes a significant difference from the control (* *p* < 0.05). SEM—standard error of the mean.

Next, we investigated the cytotoxicity of the compounds on the viability of NT2/D1 cells. Human testicular embryonal carcinoma cell line NT2/D1 was more sensitive to the tested compounds than leukemia cells (Figure 6). Our initial screening showed that Compounds 7 and 4 exhibited the strongest cytotoxic effects against NT2/D1 cells (Figure 6). Further evaluation of the cytotoxicity of these compounds at concentrations lower than 10 μM is needed to determine their IC_50_ values. Compounds 5 and 1 also showed strong cytotoxic activity with IC_50_ values of 5.5 μM and 11.2 μM (Figure 6). Compounds 2 and 3, tested for the first time in this study, exhibited similar cytotoxicity against NT2/D1 cells (15.5 μM and 6.8 μM, respectively) (Figure 6). Lack of a dose-dependent response in the treatments with these compounds suggest that further analyses of their cytotoxic activity at concentrations between 1 μM and 25 μM may contribute to the full assessment of their effect on NT2/D1 cells.

**Figure 7:**
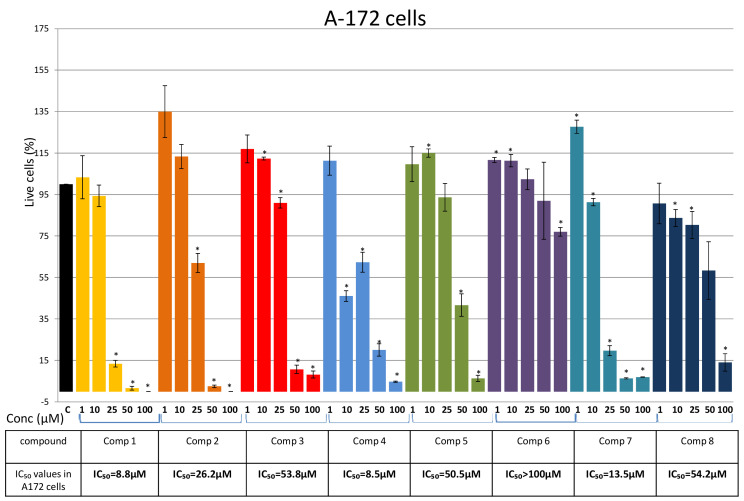
Effects of Compounds 1–8 on A-172 cells. Cells were treated for 48 h with increasing concentrations (1 μM, 10 μM, 25 μM, 50 μM, and 100 μM) of the tested compounds and cell viability was assessed using an MTT assay and expressed as the percentage of absorbance relative to the vehicle control (A-172 cells treated with DMSO), which was set at 100%. Data are presented as the mean ± SEM of at least three independent experiments. An asterisk denotes a significant difference from the control (* *p* < 0.05). SEM—standard error of the mean.

**Figure 8:**
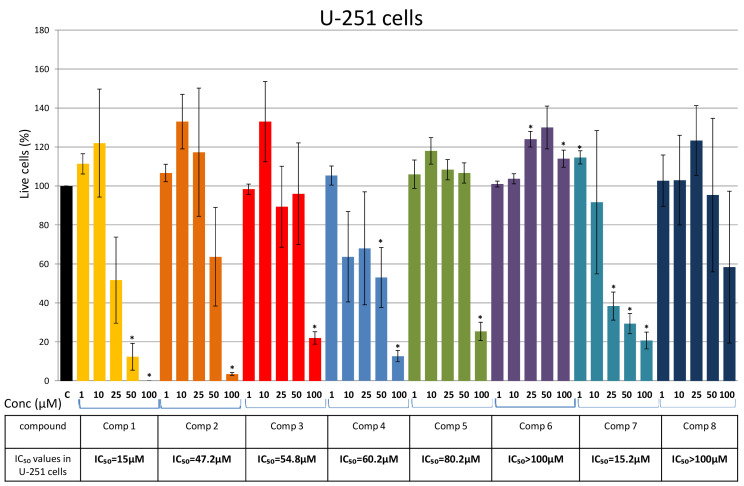
Effects of Compounds 1–8 on U-251 cells. Cells were treated for 48 h with increasing concentrations (1 μM, 10 μM, 25 μM, 50 μM, and 100 μM) of the tested compounds and cell viability was assessed using an MTT assay and expressed as the percentage of absorbance relative to the vehicle control (U251 cells treated with DMSO), which was set at 100%. Data are presented as the mean ± SEM of at least three independent experiments. An asterisk denotes significant difference from the control (* *p* < 0.05). SEM—standard error of the mean.

Having in mind that the NT2/D1 cells displayed characteristics of committed neural progenitors [21], we hypostatized that their neural origin may contribute to a similar response of the glioblastoma cells to the tested compounds. The cytotoxic effects of Compounds 1–8 on the viability of the A-172 and U251 cell lines are presented in Figure 7 and Figure 8. In A-172 cells, Compounds 4, 1, and 7 showed the highest cytotoxic effect with IC_50_ values 8.5 μM, 8.8 μM, and 13.5 μM, respectively (Figure 6). Compound 2 also exhibited a strong effect on the viability of A-172 cells (IC_50_ = 26.2 μM), while Compounds 3, 5, and 8 showed moderate cytotoxicity (Figure 6).

In U-251 cells, Compounds 1 and 7 displayed the strongest effect with IC_50_ values of ~15 μM (Figure 8). Moderate cytotoxicity was observed in treatments with Compounds 2, 3, and 4 (IC_50_ values between 40 μM and 60 μM), while Compounds 5, 6, and 8 showed low cytotoxic effects (Figure 8). A high standard error of the mean (SEM) was observed for the cytotoxic activity of Compound 1 (at concentrations of 10 μM and 25 μM), Compound 2 (at concentrations of 25 μM and 50 μM), Compound 3 and 4 (at concentrations of 10 μM, 25 μM, and 50 μM), Compound 7 (at concentrations of 10 μM), and Compound 8 (at concentrations 10 μM, 25 μM, 50 μM, and 100 μM) (Figure 8). The U-251 glioblastoma cells are endowed with glioblastoma stem cells, a population of cells with adaptive plasticity that may contribute to the heterogeneity of these cells and the differences in the responses to various stimuli [22]. We hypothesized that the variability in the U-251 cells’ sensitivity to the tested compounds reflects the heterogeneity of the population of this cell line.

Compounds 2 and 3, isolated for the first time from *P. endiviifolia*, exhibited strong to moderate effects against NT2/D1 and glioblastoma cell lines (Figure 6, Figure 7 and Figure 8). The NT2/D1 cells were more susceptible to both compounds (Figure 6).

## 3. Discussion

Perrottetin E isolated from the liverwort *Conocephalum japonicum* showed moderate cytotoxic effects against KB cells [23]. Marchantin-type bis-bibenzyls (cyclic molecules) are known in the literature as cytotoxic agents; marchantin C has been observed as cytotoxic against P388 leukemia cells, and induced angiogenic inhibition of T98G and U87 glioma cells [24,25]. Notably, microtubule-depolarizing capacity was also observed in KB, MCF-7, and PC3 cell lines of the same compound [26]. Bibenzyl lunularin isolated from the liverwort *Dumortiera hirsuta* showed moderate cytotoxicity against human HepG2 cells [27]. Cytotoxicity against chemoresistant prostate cancer PC3 cells was shown by cyclic bis-bibenzyls, riccardin C, marchantin M, and plagiochin E [28]. Molecular mechanisms of cytotoxic efficacy of bis-bibenzyls was described in terms of several biochemical markers of apoptosis and necrosis induction, such as DNA fragmentation, nuclear condensation, proteolysis of poly (ADP-ribose) polymerase (PARP), activation of caspases, inhibition of antiapoptotic nuclear transcriptional factor-kappaB, activation of p38, etc. [29].

Our results showed that the tested compounds have similar cytotoxic effects on AML and CML cell lines. Compound 7 showed the highest cytotoxicity against the U-937 and K-562 cells (IC_50_ values of 13.2 μM and 35.5 µM, respectively), while Compound 1, with an IC_50_ value of 14.2 μM, exhibited the strongest effect on the viability of HL-60 cells. Compound 2, isolated for the first time from *P. endiviifolia*, showed strong cytotoxic activity against U-937 cells (IC_50_ of 38.5 μM).

Previous studies also demonstrated the cytotoxic activity of bis-bibenzyls on K562 and HL60 leukemia cells. Riccardin D isolated from the Chinese liverwort showed the anti-proliferative effect on HL-60 and K-562 leukemia cells [27]. The IC_50_ values of riccardin D for the K562 and HL60 cells after 72 h of treatment were 21.7 μM and 25.2 μM, respectively [30]. The pro-apoptotic action of riccardin D in these cells was mediated by inhibition of topoisomerase II [30]. Shi et al. [31] examined the antiproliferative effect of plagiochin E, isolated from *Marchantia polymorpha*, on K562/A02, a multidrug-resistant counterpart of K562 cells [31]. Plagiochin E showed moderate antiproliferative effects at concentrations of 12–20 μM but it promoted sensitivity of K562/A02 cells to Adriamycin, acting as a multidrug-resistance inhibitor [31]. Dihydroptychantol A (DHA) isolated from *Asterella angusta* also increase Adriamycin cytotoxicity to K562/A02 cells [32], while its derivatives with thiazole rings showed significant anti-proliferative effects against K562 and K562/A02 cells [33]. After 48 h treatment at concentrations 20 μM, 30 μM, and 40 μM, cell viability was reduced to 58.92%, 79.26%, and 94.33%, respectively [33].

Results obtained on NT2/D1 cells showed that Compounds 1-5, and 7 exhibited strong cytotoxic effects against these cells. Further evaluation of their effects at lower concentrations is needed to fully assess their cytotoxicity against NT2/D1 cells.

To the best of our knowledge, this is the first study of the effects of bis-bibenzyls on the viability of human testicular embryonal carcinoma NT2/D1 cells. Although cells derived from human non-seminomatous testicular germ cell tumors are highly sensitive to cytoreductive agents [34], the potential therapeutic significance of bis-bibenzyls activity against this class of testicular germ cell tumors should be further investigated.

Glioblastoma cell lines also exhibited sensitiveness to the tested compounds. Compounds 4, 1, and 7 showed the highest cytotoxic effects in A-172 cells (IC_50_ values of 8.5 μM, 8.8 μM, and 13.5 μM, respectively). Similarly, in U-251 cells, Compounds 1 and 7 displayed the strongest effect with IC_50_ values of ~15 μM. Regarding the compounds isolated for the first time from *P. endiviifolia*, Compound 2 exhibited a strong effect on the viability of A-172 cells (IC_50_ = 26.2 μM) and a moderate effect on the viability of U-251 cells. Compound 3 showed moderate cytotoxicity in both glioblastoma cell lines.

Data from the literature also revealed the effects of bibenzyls on the viability of glioblastoma cell lines. The cytotoxic activity of cinnamoyl bibenzyls isolated from *Polytrichastrum pallidisetum* on U-251 (ED50 between 0.8 and 2) was previously demonstrated [35]. Macrocyclic bisbibenzyls marchantin C, isolated from *A. angusta*, at concentrations of 16 and 32 μM, showed remarkable inhibitory effects on A-172 cell viability, growth, and colony formation, by inducing apoptosis [36]. In addition, marchantin C induced significant cytotoxicity in the T98G and U87 glioblastoma cell lines at concentrations above 12 μM [25].

## 4. Materials and Methods

### 4.1. Plant Material

*Pellia endiviifolia* (Dicks.) Dumort. was collected from Bajina Basta (Serbia) in August 2018 and was identified by Prof. M. V. A voucher specimen, No. 17503, has been deposited in the Herbarium at the Institute of Botany and Botanical Garden “Jevremovac”, University of Belgrade (BEOU). The material was dried at room temperature.

### 4.2. Extract Preparation

Dried plant material was grinded into a fine powder using an electric blender. Powdered material (240 g) was extracted with a methylene-chloride/methanol 2:1 solvent system for 24 h at room temperature. After 24 h, the mixture was filtered through Whatman filter paper No. 1. The solvents were vaporized from the extract using a rotary vacuum evaporator (Laborota 4001, Heidolph, IKA, Staufen, Germany) at 40 °C. The yield of the extract was 7.5%.

### 4.3. Dry Column Flash Chromatography

The solvents used for the dry column flash chromatography were n-hexane, ethyl acetate, and methanol. At first, a gradient elution was performed using an n-hexane/EtOAc solvent system with an increasing share of EtOAc followed by an EtOAc/MeOH gradient elution with an increasing share of methanol.

### 4.4. Isolation of Bis-Bibenzyls

NMR analysis revealed two fractions of interest, those obtained by elution with 60% and 70% EtOAc in n-hexane. Both of them indicated the presence of bis-bibenzyls and were used for their isolation using semipreparative HPLC Agilent Technologies 1100 Series, Santa Clara, CA, USA, with a water/MeCN solvent system and DAD detection. The column used was a Zorbax Eclipse XDB-C18, the wavelength of detection was 280 nm, and the temperature used was 40 °C. The program used was 0–12 min 30% МеCN, and 12–25 min 30–100% MeCN. From the fraction with 60% EtOAc, perrottetin E was isolated, and from the second fraction with 70% EtOAc, two bis-bibenzyls were isolated: 10′-hydroxyperrottetin E and 10,10′-dihydroxyperrottetin E. The structure elucidation of all the isolated compounds were performed using 1D (^1^H and ^13^C) and 2D (COSY, NOESY, HSQC and HMBC) NMR spectroscopy and compared to the literature data (Figures S1–S16).

### 4.5. Cell Culture and Cytotoxicity Test

Cytotoxicity of the selected compounds was tested using three human leukemia cell lines, human embryonal teratocarcinoma cell line NT2/D1, and two human glioblastoma cell lines. Leukemia cell lines used in this study were obtained from the American Type Culture Collection (ATCC, Rockville, MD): HL-60 (acute promyelocytic leukemia cells), U-937 (acute monocytic leukemia cells), and K-562 (human chronic myelogenous leukemia cells). Human glioblastoma cell lines A-172 and U251 were also obtained from American Type Culture Collection (ATCC, Rockville, MD). Human embryonal teratocarcinoma cell line NT2/D1 was a kind gift of Prof. Paul Andrews, University of Sheffield, UK. Leukemia cells were grown in RPMI 1640 media supplemented with 10% fetal bovine serum (FBS) and an antibiotic–antimycotic solution, 100 U/mL penicillin and 100 μg/mL streptomycin (all purchased from Gibco, Thermo Fisher Scientific Inc., Waltham, MA, USA), and maintained at 37 °C in a humidified atmosphere with 5% CO_2_. NT2/D1 cells and glioblastoma cell lines A-172 and U-251 were grown in Dulbecco’s Modified Eagle Medium (DMEM)–high glucose (4500 mg/L glucose), supplemented with 10% fetal bovine serum (FBS), glutamine (2 mM), penicillin (100 U/mL), and streptomycin (100 µg/mL) (all purchased from Gibco, Thermo Fisher Scientific Inc., Waltham, MA, USA), in a humidified atmosphere with 10% CO_2_ at 37 °C. For treatments, cells were seeded in 96-well plates at a density of 2 × 10^4^ cells/well for leukemia cells, 1.5 × 10^4^ cells/well for NT2/D1, and 5 × 10^3^ cells/well for A-172 and U251. Leukemia cells, as suspension-growing cell lines, were treated immediately after seeding whereas NT2/D1, A-172, and U251 cells, adherent cells, were allowed to attach and propagate during 24 h prior to the treatment. All compounds (1–8) were initially dissolved in DMSO at a concentration of 50 mM and diluted in the cell culture medium to the desired working concentrations for the treatments (1 µM, 10 µM, 25 µM, 50 µM, and 100 µM). Corresponding dilutions of DMSO in tissue culture medium were used as a vehicle-treated controls. The final concentration of DMSO in the culture medium did not exceed 0.2%.

Following 48 h of treatment, cell viability was determined using colorimetric assays for quantification of viable cells. For leukemia cells, an MTS [3-(4,5-dimethylthiazol-2-yl)-5-(3-carboxymethoxyphenyl)-2-(4-sulfophenyl)-2H-tetrazolium)] assay was used according to the manufacturer’s instructions (Promega, Madison, WI, USA). For NT2/D1, A-172, and U251 cells, the cytotoxicity was measured by an MTT [3-(4,5-dimethylthiazol-2-yl)-2,5-diphenyltetrazolium bromide] assay. After treatment, cells were incubated with an MTT solution (MTT (Merck KGaA, Darmstadt, Germany) dissolved in a cell culture medium to a final concentration of 0.5 mg/mL) for 4 h. After incubation, the medium was removed and the formazan crystals were dissolved in DMSO (dimethyl sulfoxide) (SERVA Electrophoresis GmbH, Heidelberg, Germany).

Absorbance was measured at 490 nm using a Tecan Infinite 200 Pro multiplate reader (Tecan Group, Mannedorf, Switzerland). All experiments were performed in triplicates, repeated at least three times. The relative cell viability (%) of the treated cells was calculated as a percentage of the vehicle control (cells treated with DMSO) set to 100%. Data were analyzed using IBM SPSS Statistics and GraphPad Prism.

## 5. Conclusions

The present study demonstrated the chemical constituents of *P. endiviifolia* liverwort and the cytotoxic activity of its perrottetin-type compounds. Additionally, our results suggest that all compounds tested exhibited a cytotoxic effect. The most active compound from *P. endiviifolia* was perrottetin E (**1**), while, overall, the most active was perrottetin F phenanthrene derivative (**7**), added to this investigation from our previous study for comparison. Future studies should be performed in the direction of investigating the cytotoxic mechanisms of bis-bibenzyls. Overall, this work increased the knowledge of the cytotoxic potential of perrottetin-type compounds and their further application as potential anticancer drugs.

## Supplementary Materials

The following are available online at https://www.mdpi.com/article/10.3390/plants10061063/s1. Figure S1: Part of the 1H NMR spectrum of 1 (perrottetin E) from δH 6.50–7.30 ppm; Figure S2: Part of the 13C NMR spectrum of 1 (perrottetin E) from δC 112–158 ppm; Figure S3: Part of the 13C NMR spectrum of 1 (perrottetin E) from δC 36.7–38.0 ppm; Figure S4: Part of the HSQC spectrum of 1 (perrottetin E) from δH 6.50–7.20 ppm and δC 112–130 ppm; Figure S5: Part of the HMBC spectrum of 1 (perrottetin E) from δH 6.50–7.2 ppm and δC 112–160 ppm; Figure S6: Part of the 1H NMR spectrum of 2 (10′-hydroxyperrottetin E) δH 6.60–7.20 ppm; Figure S7: Part of the 13C NMR spectrum of 2 (10′-hydroxyperrottetin E) from δC 112–156 ppm; Figure S8: Part of the 13C NMR spectrum of 2 (10′-hydroxyperrottetin E) from δC 32-38 ppm; Figure S9: Part of the COSY spectrum of 2 (10′-hydroxyperrottetin E) from δH 6.50–7.30 ppm; Figure S10: Part of the HSQC spectrum of 2 (10′-hydroxyperrottetin E) from δH 6.55–7.20 ppm and δC 112–131 ppm; Figure S11: Part of the HMBC spectrum of 2 (10′-hydroxyperrottetin E) from δH 6.55–7.20 ppm and δC 112–156 ppm; Figure S12: Part of the 1H NMR spectrum of 3 (10,10′-dihydroxyperrottetin E) from δH 4.80–7.20 ppm; Figure S13: Part of the 13C NMR spectrum of 3 (10,10′-dihydroxyperrottetin E) from δC 112–157 ppm; Figure S14: Part of the COSY spectrum of 3 (10,10′-dihydroxyperrottetin E) from δH 6.50–7.30 ppm; Figure S15.: Part of the HSQC spectrum of 3 (10,10′-dihydroxyperrottetin E) from δH 6.40–7.15 ppm and δC 112–130 ppm; Figure S16: Part of the HMBC spectrum of 3 (10,10′-dihydroxyperrottetin E) from δH 6.40–7.15 ppm and δC 112–156 ppm.
