# Supplementary material for: Bis-Bibenzyls from the Liverwort Pellia endiviifolia and Their Biological Activity
Source: Plants (Basel). 2021 May 26;10(6):1063. doi: 10.3390/plants10061063 (PMC8227020; doi:10.3390/plants10061063)
Supplement: Supplementary file 1 [file plants-10-01063-s001.zip › plants-1206356-supplementary.pdf]

Supplementary Material

Bis-bibenzyls from the liverwort *Pellia endiviifolia* and their biological activity

Ivana Ivković[1], Miroslav Novaković[2], Milan Veljić[1], Marija Mojsin[3], Milena Stevanović[3,4,5], Petar D. Marin[1], Danka Bukvički[1,]*

[1]*University of Belgrade, Faculty of Biology, Institute of Botany and Botanical Garden "Jevremovac", 11000 Belgrade, Serbia.*

[2]*University of Belgrade, Institute of Chemistry Technology and Metallurgy, Department of Chemistry, 11 000 Belgrade, Serbia.*

[3]*University of Belgrade, Institute of Molecular Genetics and Genetic Engineering, 11042 Belgrade, Serbia.*

[4]*University of Belgrade, Faculty of Biology, 11000 Belgrade, Serbia.*

[5]*Serbian Academy of Sciences and Arts, 11001 Belgrade, Serbia.*

*Author to whom correspondence should be addressed:

Dr Danka Bukvicki;
e-mails: dankabukvicki@gmail.com, dankabukvicki@bio.bg.ac.rs,
telephone: +381-63-161-1980;
fax: +38-111-324-3603.

Figure S1. Part of the ^{1}H NMR spectrum of **1** (perrottetin E) from δ_H 6.50-7.30 ppm

Figure S2. Part of the ^{13}C NMR spectrum of **1** (perrottetin E) from δ_C 112-158 ppm

Figure S3. Part of the ^{13}C NMR spectrum of **1** (perrottetin E) from δ_C 36.7-38.0 ppm

Figure S4. Part of the HSQC spectrum of **1** (perrottetin E) from δ_H 6.50-7.20 ppm and δ_C 112-130 ppm

Figure S5. Part of the HMBC spectrum of **1** (perrottetin E) from δ_H 6.50-7.2 ppm and δ_C 112-160 ppm

Figure S6. Part of the ^{1}H NMR spectrum of **2** (10′-hydroxyperrottetin E) δ_H 6.60-7.20 ppm

Figure S7. Part of the ^{13}C NMR spectrum of **2** (10′-hydroxyperrottetin E) from δ_C 112-156 ppm

Figure S8. Part of the ^{13}C NMR spectrum of **2** (10′-hydroxyperrottetin E) from δ_C 32-38 ppm

Figure S9. Part of the COSY spectrum of **2** (10′-hydroxyperrottetin E) from δ_H 6.50-7.30 ppm

Figure S10. Part of the HSQC spectrum of **2** (10′-hydroxyperrottetin E) from δ_H 6.55-7.20 ppm and δ_C 112-131 ppm

Figure S11. Part of the HMBC spectrum of **2** (10′-hydroxyperrottetin E) from δ_H 6.55-7.20 ppm and δ_C 112-156 ppm

Figure S12. Part of the ^{1}H NMR spectrum of **3** (10,10′-dihydroxyperrottetin E) from δ_H 4.80-7.20 ppm

Figure S13. Part of the ^{13}C NMR spectrum of **3** (10,10′-dihydroxyperrottetin E) from δ_C 112-157 ppm

Figure S14. Part of the COSY spectrum of **3** (10,10′-dihydroxyperrottetin E) from δ_H 6.50-7.30 ppm

Figure S15. Part of the HSQC spectrum of **3** (10,10′-dihydroxyperrottetin E) from δ_H 6.40-7.15 ppm and δ_C 112-130 ppm

Figure S16. Part of the HMBC spectrum of **3** (10,10′-dihydroxyperrottetin E) from δ_H 6.40-7.15 ppm and δ_C 112-156 ppm

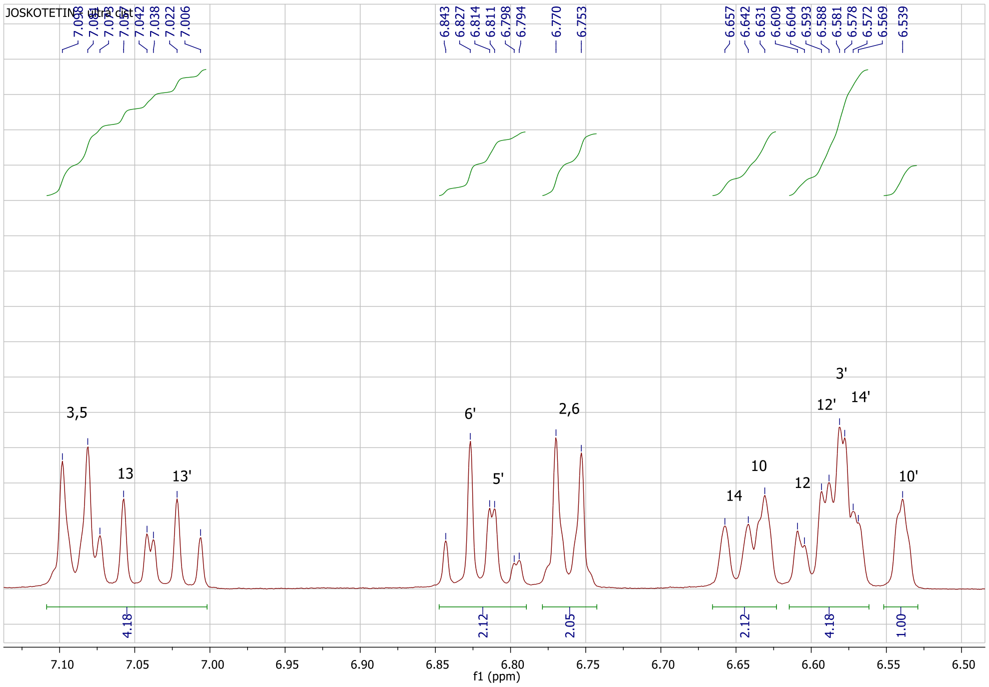

Figure S1. Part of the ^{1}H NMR spectrum of **1** (perrottetin E) from δ_H 6.50-7.30 ppm

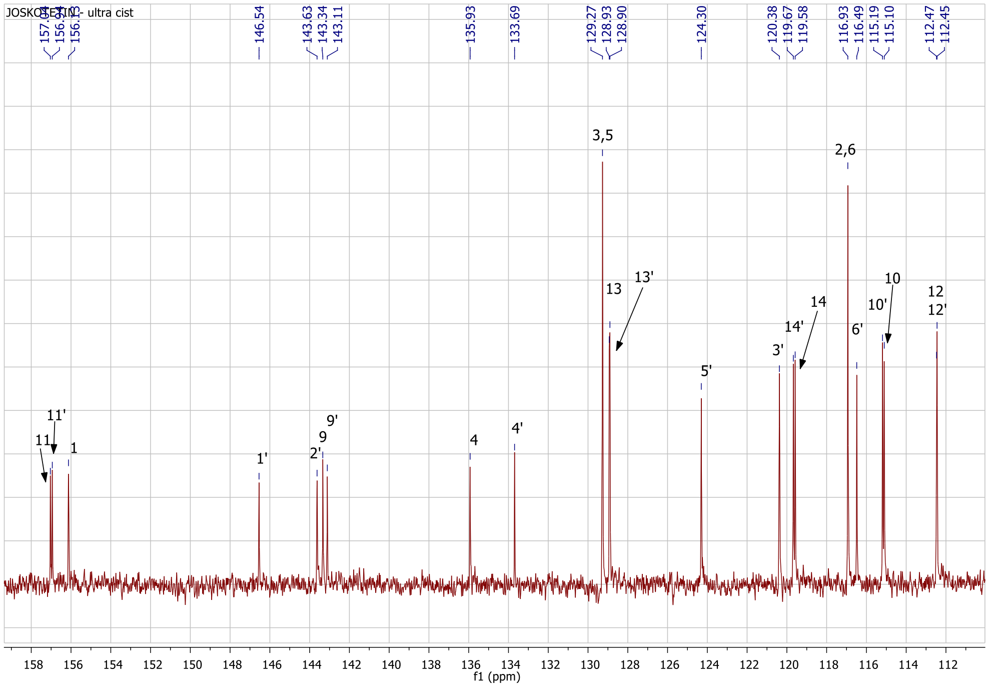

Figure S2. Part of the ^{13}C NMR spectrum of **1** (perrottetin E) from δ_C 112-158 ppm

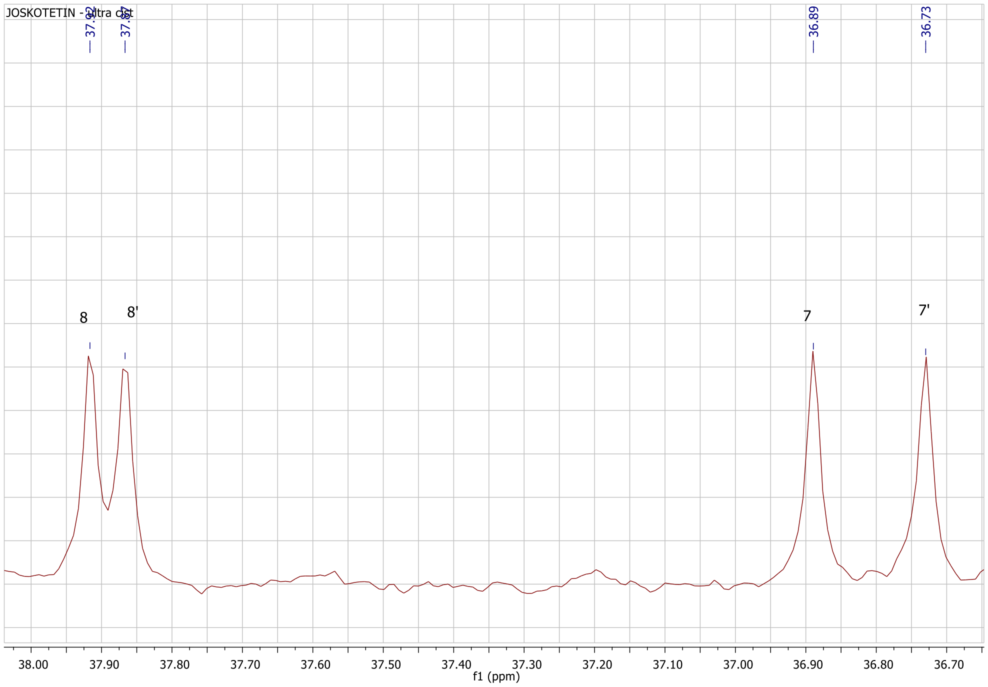

Figure S3. Part of the ^{13}C NMR spectrum of **1** (perrottetin E) from δ_C 36.7-38.0 ppm

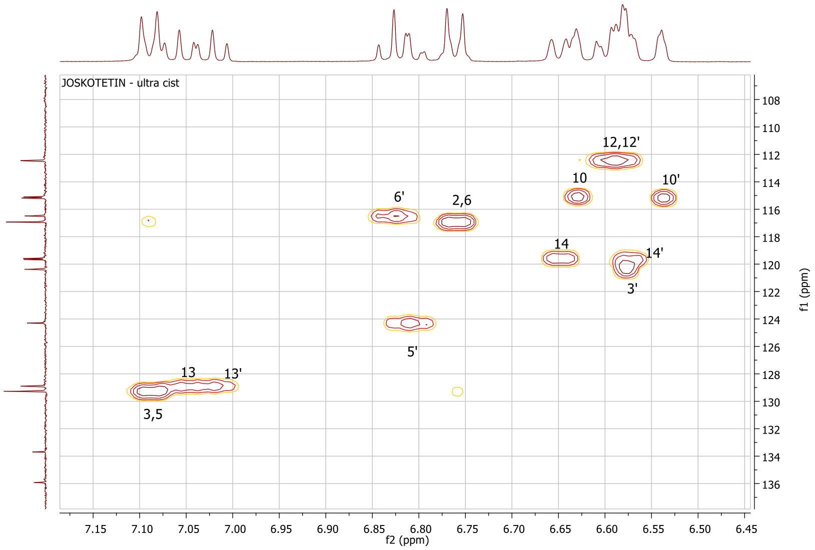

Figure S4. Part of the HSQC spectrum of **1** (perrottetin E) from δ_H 6.50-7.20 ppm and δ_C 112-130 ppm

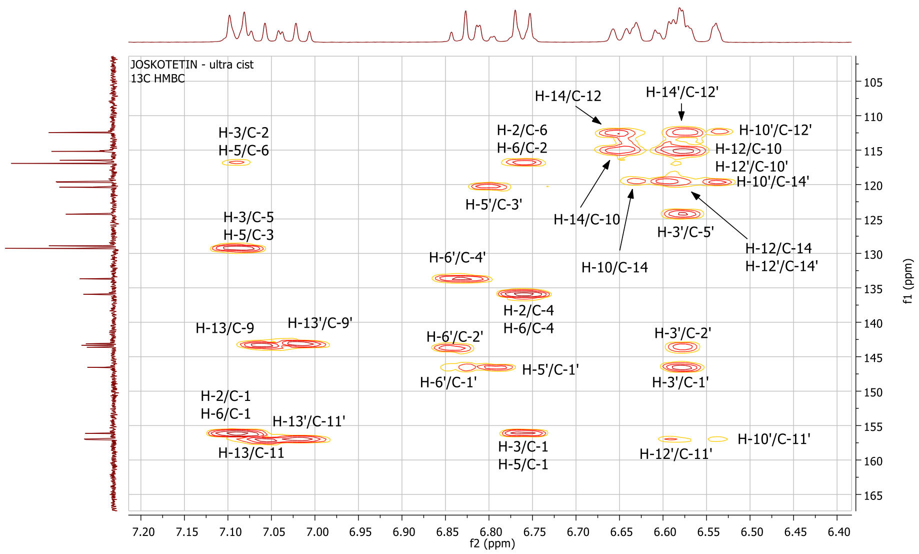

Figure S5. Part of the HMBC spectrum of **1** (perrottetin E) from δ_H 6.50-7.2 ppm and δ_C 112-160 ppm

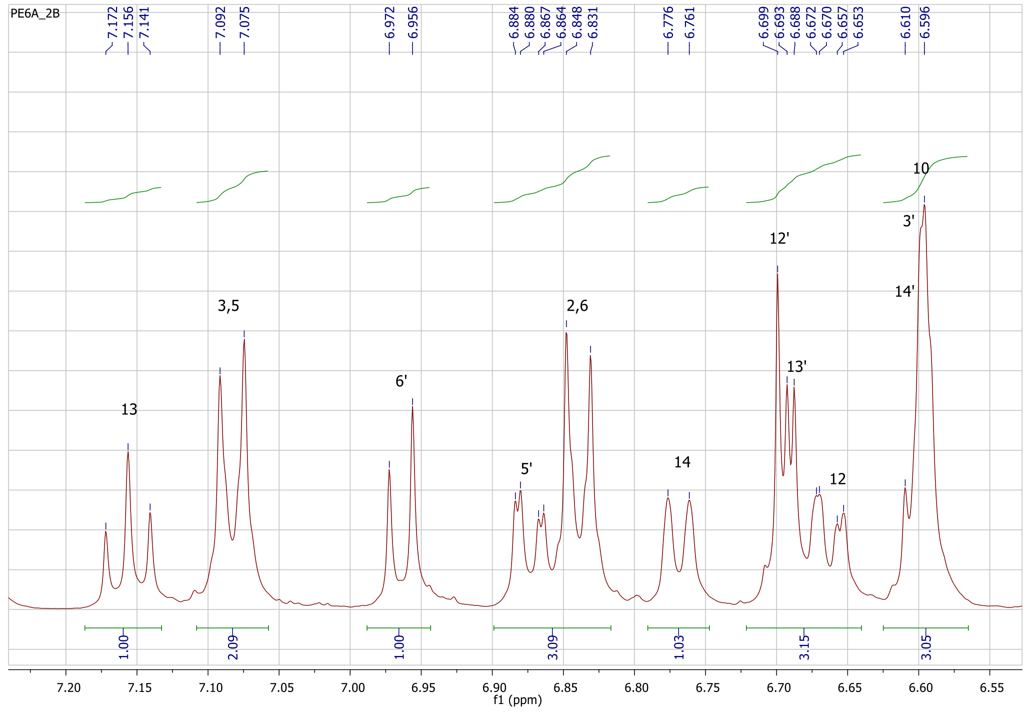

Figure S6. Part of the ^{1}H NMR spectrum of **2** (10′-hydroxyperrottetin E) δ_H 6.60-7.20 ppm

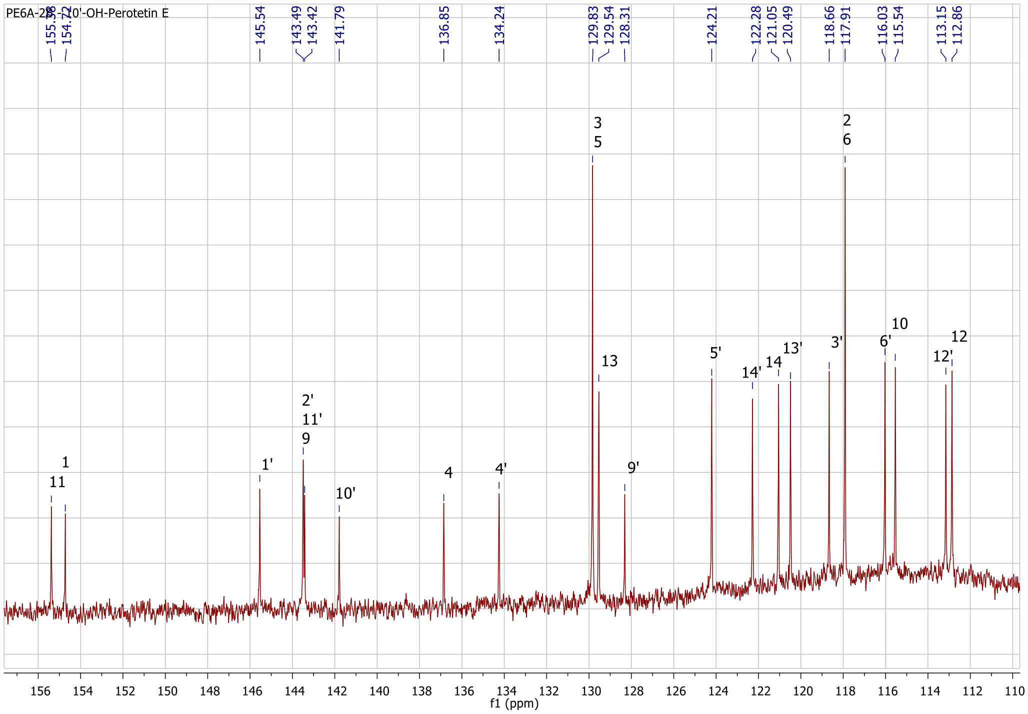

Figure S7. Part of the ^{13}C NMR spectrum of **2** (10′-hydroxyperrottetin E) from δ_C 112-156 ppm

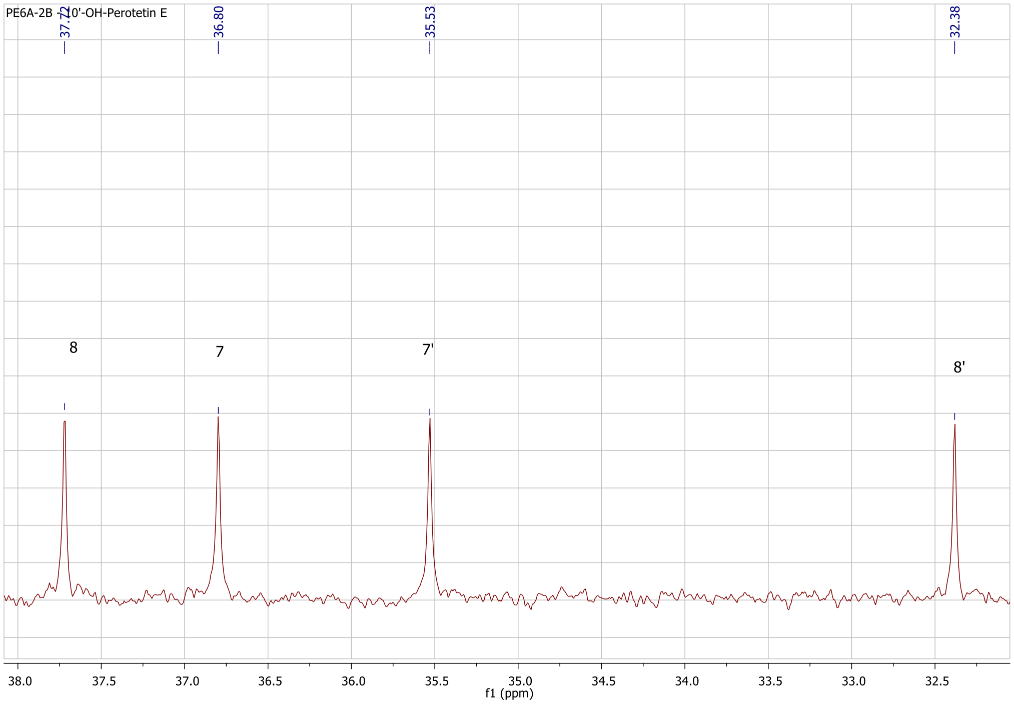

Figure S8. Part of the ^{13}C NMR spectrum of **2** (10′-hydroxyperrottetin E) from δ_C 32-38 ppm

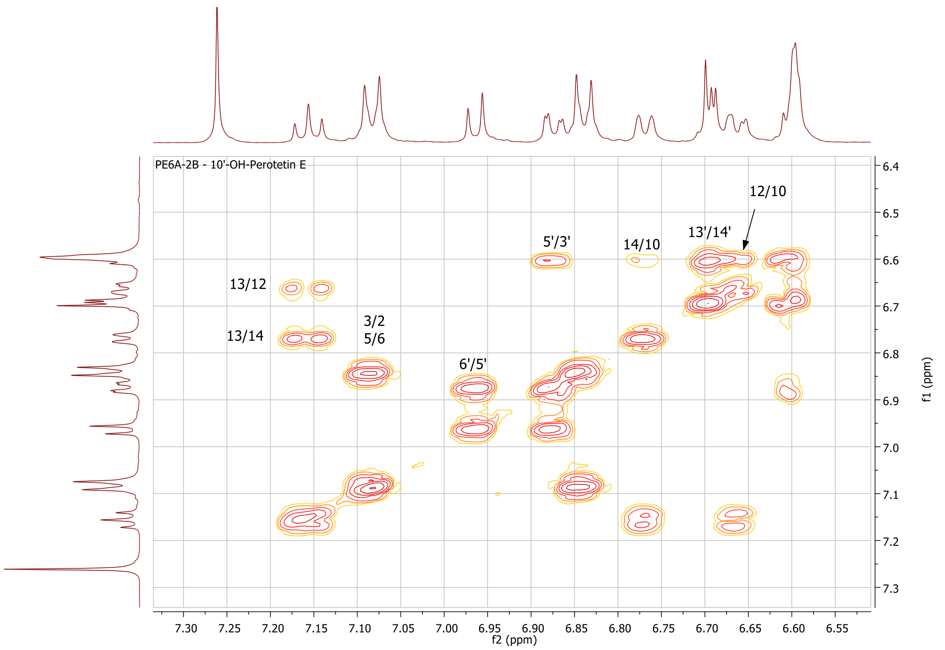

Figure S9. Part of the COSY spectrum of **2** (10′-hydroxyperrottetin E) from δ_H 6.50-7.30 ppm

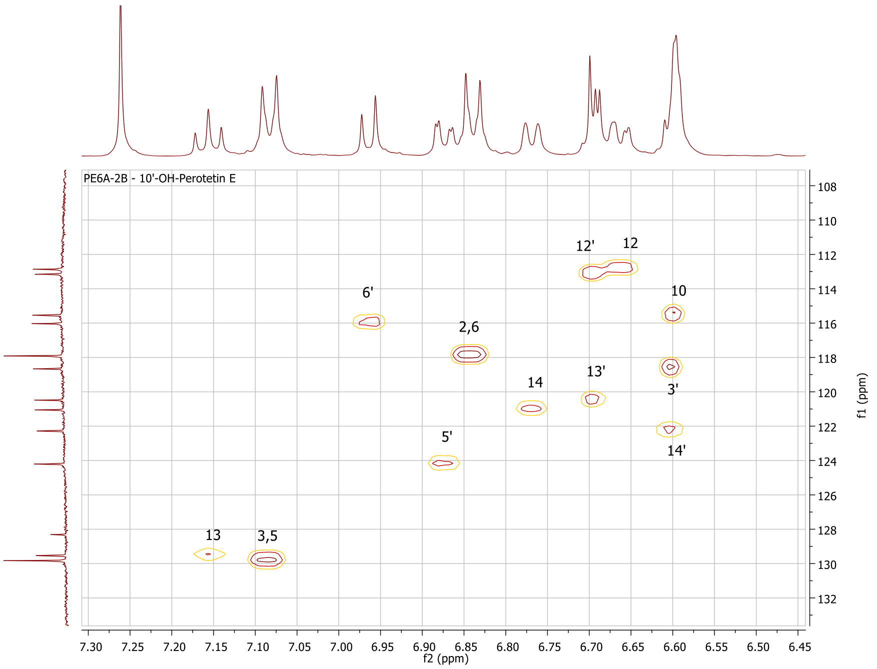

Figure S10. Part of the HSQC spectrum of **2** (10′-hydroxyperrottetin E) from δ_H 6.55-7.20 ppm and δ_C 112-131 ppm

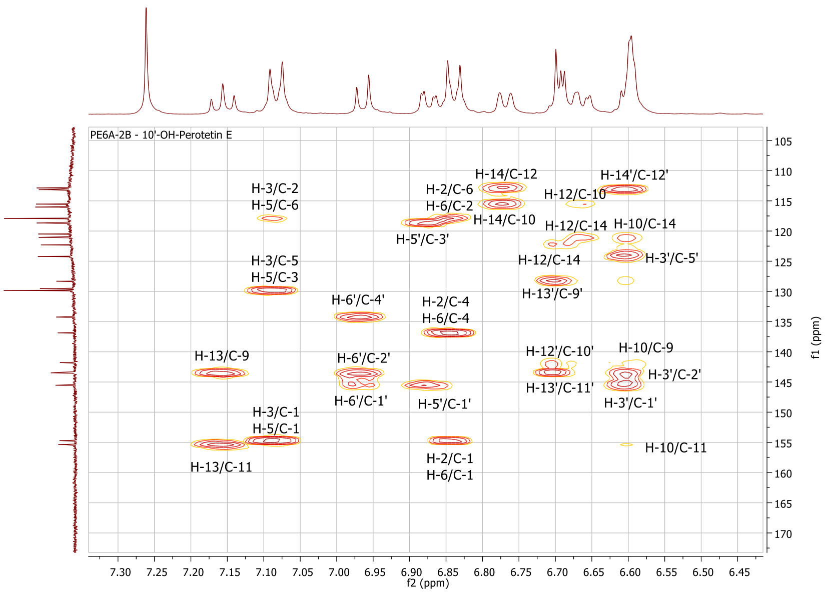

Figure S11. Part of the HMBC spectrum of **2** (10′-hydroxyperrottetin E) from δ_H 6.55-7.20 ppm and δ_C 112-156 ppm

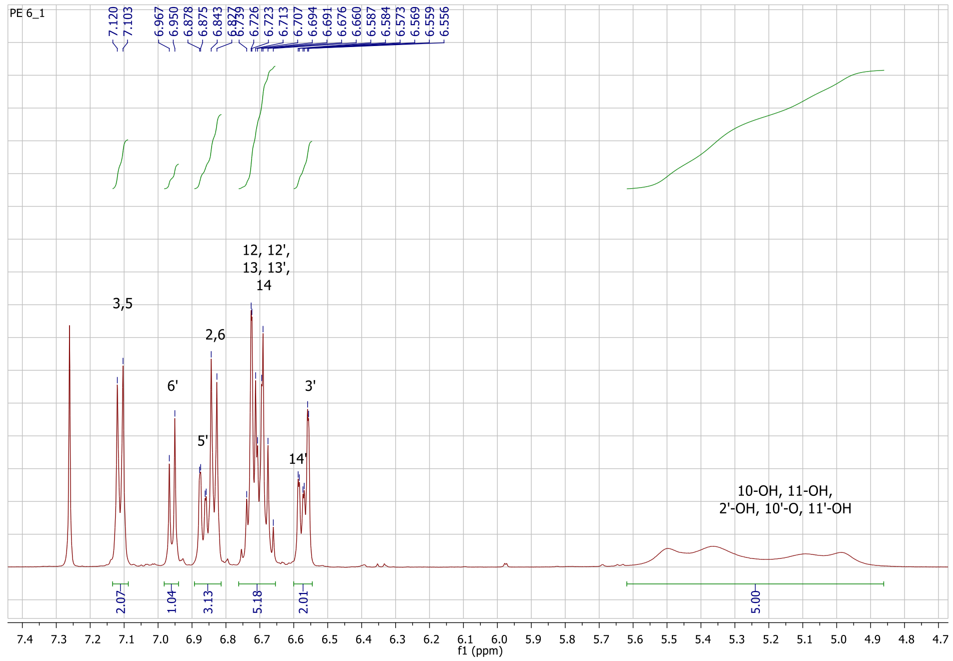

Figure S12. Part of the ^{1}H NMR spectrum of **3** (10,10′-dihydroxyperrottetin E) from δ_H 4.80-7.20 ppm

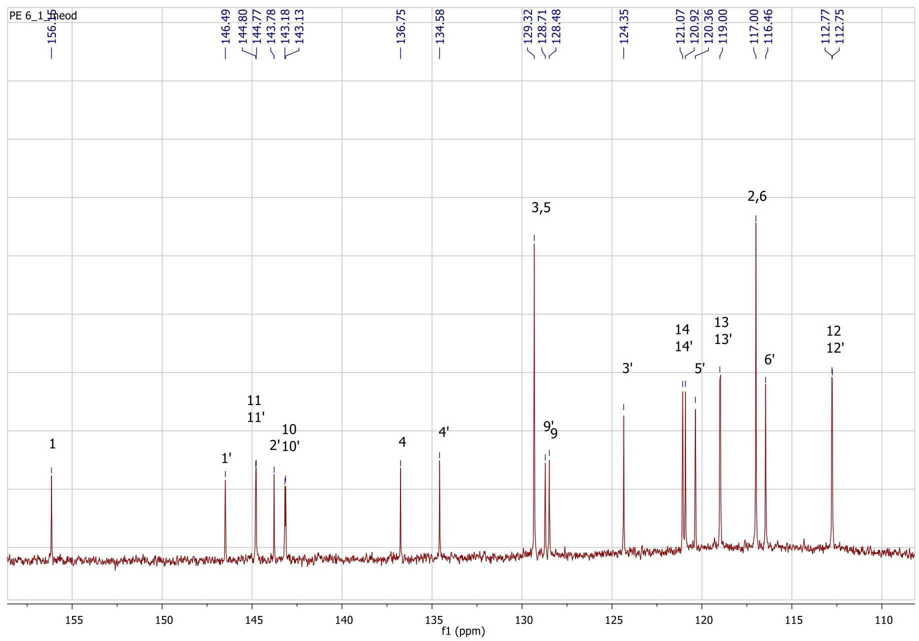

Figure S13. Part of the ^{13}C NMR spectrum of **3** (10,10′-dihydroxyperrottetin E) from δ_C 112-157 ppm

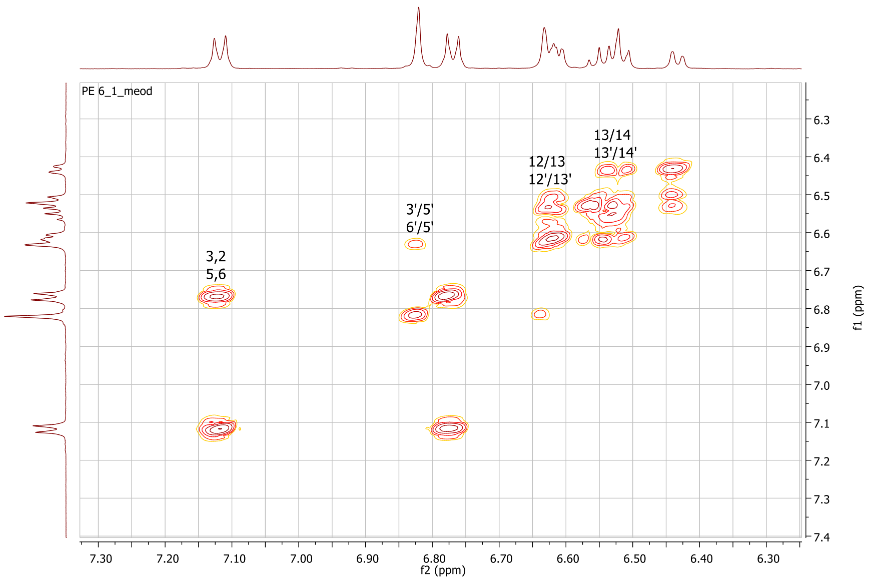

Figure S14. Part of the COSY spectrum of **3** (10,10′-dihydroxyperrottetin E) from δ_H 6.50-7.30 ppm

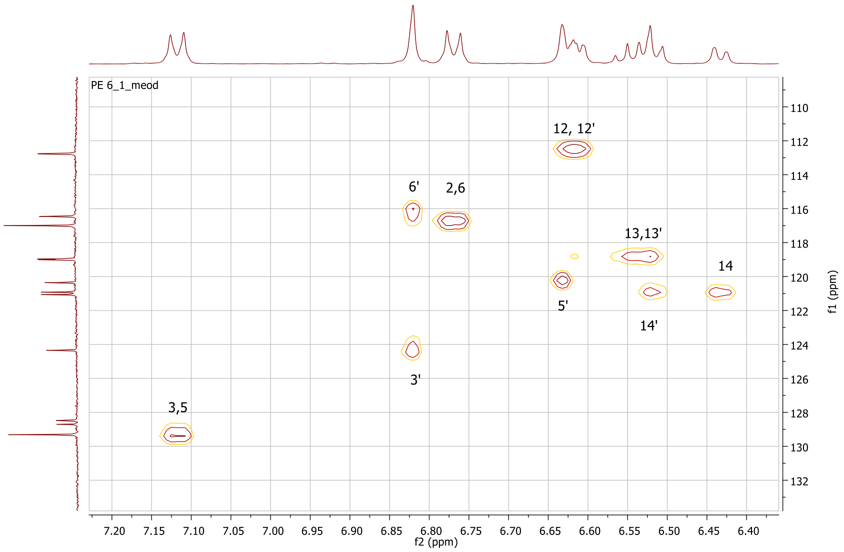

Figure S15. Part of the HSQC spectrum of **3** (10,10′-dihydroxyperrottetin E) from δ_H 6.40-7.15 ppm and δ_C 112-130 ppm

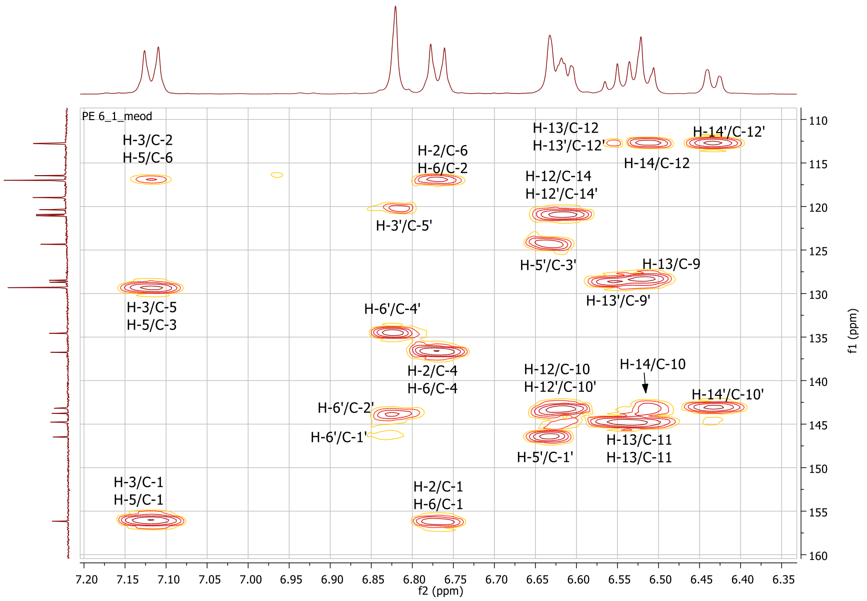

Figure S16. Part of the HMBC spectrum of **3** (10,10′-dihydroxyperrottetin E) from δ_H 6.40-7.15 ppm and δ_C 112-156 ppm
